# Novel paradigm enables accurate monthly gestational screening to prevent congenital toxoplasmosis and more

**DOI:** 10.1101/2023.04.26.23289132

**Published:** 2023-04-26

**Authors:** Y Zhou, K Leahy, A Grose, J Lykins, M Siddiqui, N Leong, P Goodall, S Withers, K Ashi, S Schrantz, V Tesic, A P Abeleda, K Beavis, F Clouser, M Ismail, M Christmas, R Piarroux, D Limonne, E Chapey, S Abraham, I Baird, J Thibodeau, K Boyer, E Torres, S Conrey, K Wang, MA Staat, N Back, J Gomez Marin, F Peyron, S Houze, M Wallon, R McLeod

**Affiliations:** 1 The University of Chicago Medicine, Chicago, Illinois (Pritzker School of Medicine; [AG,KA,RM], Pediatrics{RM], OVS[RM.YZ], Pathology[VT;KB;AA], Medicine[SS];The College [RM,JT, IB], Global Health Center [RM;JT,IJB]); 2 LDBioDiagnostics, Lyon, France ; Current address : RPiarroux, Efor CVO, champagne aux monts d’or, France; 3 Institut des agents infectieux, Hôpital de la Croix-Rousse, Lyon, France; 4 Bichat-Claude Bernard Hôpital, Laboratory of Parasitologie, Paris, France; 5 Rush Presbyterian Hospital and Medical Center, Chicago, Illinois; 6 University of Quindio, Quindio, Armenia, Colombia; 7 University of Cincinnati and Cincinnati Children’s Hospital Medical Center, Division of Infectious Diseases; University of Cincinnati, Department of Pediatrics (MAS)., Cincinnati, Ohio

## Abstract

**Background:**

Congenital toxoplasmosis is a treatable, preventable disease, but untreated causes death, prematurity, loss of sight, cognition and motor function, and substantial costs worldwide.

**Methods/ Findings.:**

In our ongoing USA feasibility/efficacy clinical trial, data collated with other ongoing and earlier published results proved high performance of an Immunochromatographic-test(ICT) that enables accurate, rapid diagnosis/treatment, establishing new paradigms for care. Overall results from patient blood and/or serum samples tested with ICT compared with gold-standard-predicate-test results found ICT performance for 4606 sera/1 876 blood, 99.3%/97.5% sensitive and 98.9%/99.7% specific. However, in the clinical trial the FDA-cleared-predicate test initially caused practical, costly problems due to false-positive-IgM results. For 58 persons, 3/43 seronegative and 2/15 chronically infected persons had false positive IgM predicate tests. This caused substantial anxiety, concerns, and required costly, delayed confirmation in reference centers. Absence of false positive ICT results contributes to solutions: Lyon and Paris France and USA Reference laboratories frequently receive sera with erroneously positive local laboratory IgM results impeding patient care. Therefore, thirty-two such sera referred to Lyon’s Reference laboratory were ICT-tested. We collated these with other earlier/ongoing results: 132 of 137 USA or French persons had false positive local laboratory IgM results identified correctly as negative by ICT. Five false positive ICT results in Tunisia and Marseille, France, emphasize need to confirm positive ICT results with Sabin-Feldman-Dye-test or western blot. Separate studies demonstrated high performance in detecting acute infections, meeting FDA, CLIA, WHO ASSURED, CEMark criteria and patient and physician satisfaction with monthly-gestational-ICT-screening.

**Conclusions/Significance.:**

This novel paradigm using ICT identifies likely false positives or raises suspicion that a result is truly positive, rapidly needing prompt follow up and treatment. Thus, ICT enables well-accepted gestational screening programs that facilitate rapid treatment saving lives, sight, cognition and motor function. This reduces anxiety, delays, work, and cost at point-of-care and clinical laboratories.

## Author’s Summary

Toxoplasmosis is a major health burden for developed and developing countries, causing damage to eyes and brain, loss of life and substantial societal costs. Prompt diagnosis in gestational screening programs enables treatment, thereby relieving suffering, and leading to > 14-fold cost savings for care. Herein, we demonstrate that using an ICT that meets WHO ASSURED-criteria identifying persons with/without antibody to *Toxoplasma gondii* in sera and whole blood with high sensitivity and specificity, is feasible to use in USA clinical practice. We find this new approach can help to obviate the problem of detection of false positive anti-*T.gondii* IgM results for those without IgG antibodies to *T.gondii* when this occurs in present, standard of care, predicate USA FDA cleared available assays. Thus, this accurate test facilitates gestational screening programs and a global initiative to diagnose and thereby prevent and treat *T.gondii* infection. This minimizes likelihood of false positives (IgG and/or IgM) while maintaining maximum sensitivity. When isolated IgM antibodies are detected, it is necessary to confirm and when indicated continue follow up testing in ~2 weeks to establish seroconversion. Presence of a positive ICT makes it likely that IgM is truly positive and a negative ICT makes it likely that IgM will be a false positive without infection. These results create a new, enthusiastically-accepted, precise paradigm for rapid diagnosis and validation of results with a second-line test. This helps eliminate alarm and anxiety about false-positive results, while expediting needed treatment for true positive results and providing back up distinguishing false positive tests.

## INTRODUCTION

*Toxoplasma gondii*, infects approximately half of all persons with 16 million people infected congenitally. Congenital toxoplasmosis (CT) causes loss of life, sight, cognitive and motor function [1-5]. In 2013 the World Health Organization (WHO) estimated there are up to 190,100 new cases of CT and 1.20 million disability-adjusted life years each year globally [4-7]. Disease burden is particularly high in Latin America and certain populations in the US and elsewhere with high exposure. Almost all untreated congenitally infected persons develop manifestations [1-17]. In addition to considerable progress toward definitive cure and prevention of *Toxoplasma* infection with novel potential medicines and vaccines [6, 7], a critical part of eliminating the disease and reducing suffering and disease burden of CT requires prompt recognition of seroconversion and expeditious, early treatment of the acutely infected pregnant women with available, effective medicines [3,6-9]. Screening monthly, beginning before or near conception to one month post-partum for development of antibody to the parasite in previously seronegative women can enable treatment to prevent transplacental transmission of newly acquired maternal *Toxoplasma* infection or treat the fetus to prevent sequelae [3,7-17]. France, Austria, Slovenia, Colombia, Panama, Brazil, Argentina and Morocco have or are developing screening programs [8,10, 11] but the United States does/has not [3,6,7,8,12-14]. Actual and artificially inflated costs to make profit are potential barriers [1, 13-45], even though cost benefit analyses all have found substantial cost savings and benefits with routine testing [13-18]. Introduction of prenatal screening tests that fulfill the WHO ASSURED criteria (Affordable, Sensitive, Specific, User-friendly, Rapid, Robust, Equipment-free, Deliverable) can improve benefit [13-18]. False positive results using currently available commercial test kits for anti-*Toxoplasma* IgM compound problems [19-22, 38]. The United States FDA mandated that a positive result for acute infection (IgM) with a non-reference laboratory (NRL) test should be confirmed at the Palo Alto Medical Foundation /Remington Specialty *Toxoplasma* Serology Laboratory (PAMF-TSL) [22]. Associated delays cause concern for patients and their physicians. Substantial costs (more than $800 per panel of tests in the USA with additional problems for this exceptional testing from insurance denials and capitation of obstetrical health care) have been an argument against screening programs [14]. Therefore, NRL tests with high specificity and low cost are needed. A recently developed *Toxoplasma* ICT IgG-IgM test (LDBIO Diagnostic, Lyon, France, hereafter called ICT) is a promising candidate NRL test that satisfies ASSURED criteria [23-5].

To begin to implement a reasonable, feasible, low cost workflow for USA gestational screening programs, which has currently and historically been problematic (please see **Commentary** in supplemental showing those problems for care), a series of studies described herein were performed. A formal clinical trial feasibility study at the University of Chicago Medical Center (UCMC) began in July 2020. The goal was to evaluate a sufficient number of verifiable ICT results to complete the U.S. Food and Drug Administration (FDA) 510(k) clearance and Clinical Laboratory Improvement Amendments (CLIA) regulations waiver process. This study involves comparing results of the ICT to an already cleared serum test, also called predicate test (Bio-Rad Platelia Toxo Enzyme Immunoassay). When we encountered difficulties with false positive IgM results with the predicate comparator, but not the ICT, we were faced with the unanticipated constraint of cost of positive confirmation of a number of tests at PAMF-TSL, and recognition that this type of cost from the frequent false positive IgM results could de-rail screening programs in the United States. Given the true negative with ICT in our setting, we queried whether the ICT could be part of a paradigm to rule-out false positive IgM results with NRL test, both at the point-of-care and in the hospital clinical laboratory. Further, we tested samples with the ICT that were suspected false positives from local laboratories that had been referred to reference laboratories. We placed these data in the context of practical clinical problems we encountered and collated our results with ongoing and reported similar studies to define whether this could be a paradigm helpful in addressing false positive predicate NRL test results. Solving this problem emphasized how the ICT can be used in screening programs to benefit pregnant women and their families, creating a new paradigm to approach the problem of need for accurate screening and of false positive tests. This highly accurate test may help enable screening for acquisition of *T. gondii* in gestation [23-39] and thereby contribute to saving sight, cognition, motor function and lives and improve quality of life [1-10, 12-15, 17, 24, 35].

## METHODS

### Hypothesis

**Figure 1.**
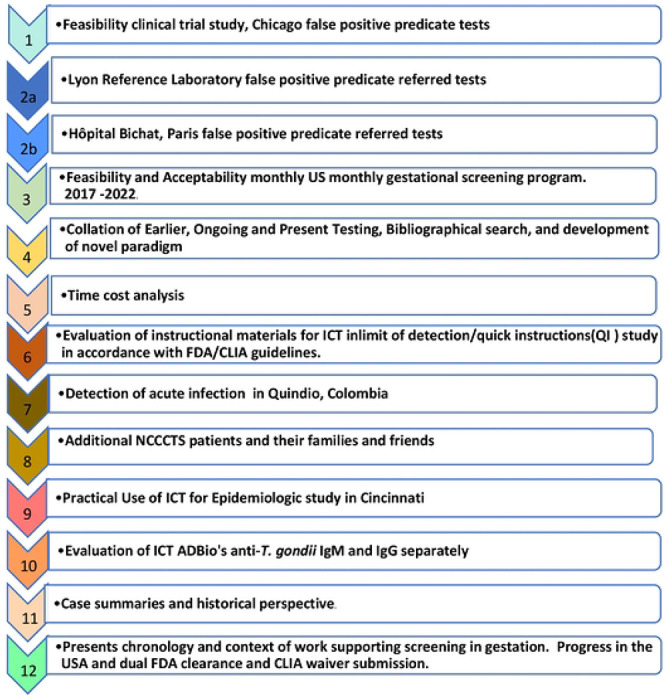
“Roadmap” to Studies Performed herein. Key shows corresponding Supplemental figures and tables.

Our hypothesis was that a lateral immunochromatography test (ICT) we previously found sensitive and specific could meet criteria specified by USA FDA and CLIA to document that this test is useful for serologic and whole blood point of care testing to detect *Toxoplasma* infection. We performed a series of studies (Figure 1) to test this hypothesis in the USA, France, and Colombia. In so doing, we discovered paradigm shifting approaches and utility, proving and extending beyond our original hypothesis, in studies using methods that follow and in the supplement in more detail. Figure 1 lists and provides a “roadmap” to 12 studies/analyses in this decades-long work. A succinct overview of methods follows:

**Table:** Key^a^

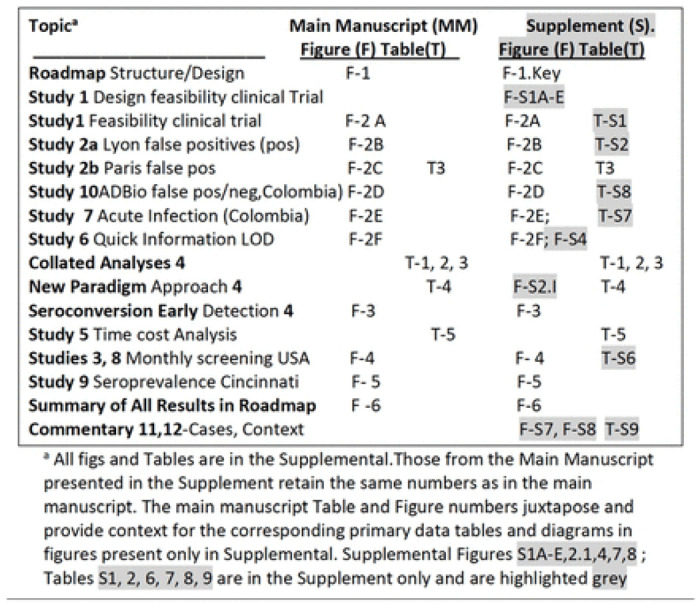

### Study 1: Feasibility clinical trial study

The design of this study (ClinicalTrials.gov number NCT04474132) and how it is related to earlier work is shown in **Supplemental Figure S1A-E**. Serologic samples for the UCMC feasibility study (ongoing as of April 2023) were obtained from 41 pregnant women, 40 undergoing regular prenatal appointments at the UCMC (23 in first trimester, 12 in second trimester, four in third-trimester) and from seventeen non-pregnant volunteers. Each subject’s whole blood and sera were tested with ICT; subjects’ sera were also tested at the UCMC’s CLIA-approved Clinical Laboratory, which uses a Bio-Rad Platelia Toxo Enzyme Immunoassay as its FDA-cleared standard predicate test to detect IgG and IgM *Toxoplasma* antibodies. There were three testers in 3 sites. All discrepant results between ICT and predicate were sent to Remington Specialty Laboratory PAMF-TSF or Lyon Reference laboratory for confirmation immediately using a panel of tests described elsewhere [27, 37].

### Study 2a: Additional samples from Lyon Reference Laboratory that had been referred when erroneously reported/referred by local laboratories with positive IgM

A set of 32 samples obtained at the Parasitology Laboratory of the University Hospital of Lyon, France (Institut des agents infectieux, Hôpital de la Croix-Rousse, Lyon, France) were selected for being reported as false positive with at least one first line, NRL automated assay and confirmed to be negative by a panel of additional tests in the laboratory. They were additionally tested at LDbio Diagnostics using ICT and WB ToxoII IgG and IgM [40-45].

### Study 2b. Testing of other samples at Hôpital Bichat, Paris

A total of 558 US serum samples that would otherwise be discarded were tested at Hôpital Bichat.,Paris (Bichat-Claude Bernard Hôpital, Laboratory of Parasitologie, Paris, France [30]). Another set of samples also was tested at Hôpital Bichat in Paris. Results are being presented in detail in a separate report describing a variety of tests from Hôpital Bichat (Abraham et al, 30, and in submission 2023).

### Study 3: Testing of US Samples in a study to Determine Feasibility and Acceptability of fingerstick in monthly US gestational screening program 2017 -2022

This separate study was to determine whether this ICT testing could be performed monthly for pregnant women in an academic obstetrical setting in the USA and whether it would be acceptable for patients and their physicians. This study took place in the outpatient Obstetrics and Gynecology Practice at an urban academic medical center between September, 2017 and September 2018. Patients were identified at their first outpatient obstetrical visit, between 8-12 weeks gestation, by their primary obstetrical care provider. Patients not infrequently attended their obstetrical visit with their partners. They were provided an educationl pamphlet [33] and were able to ask any questions. Patients then were offered an opportunity to participate in the monthly screening study and if they wished to do so to sign an informed consent. If the patient indicated interest in participating in the study, voluntary consent was obtained by the research team. All patients who were asked expressed interest and willingness to participate. The original intent of the study was to follow 20 women to term with monthly testing through the sixth week post-partum obstetrical visit. Each month, at the patients’ regularly scheduled appointment or shortly thereafter the patient was tested with the whole blood-variant *Toxoplasma* ICT IgG- IgM POC test. Methods for testing have been discussed in our previous work [23] and above. Serum was tested with another high-functioning test, i.e.,with the ARCHITECT-, and /or VIDAS (VITEK^®^ ImmunoDiagnostic Assay System) as an automated enzyme-linked fluorescent immunoassay (ELFA) and/or Western Blot-Toxo-IgG and IgM systems (LDBio diagnostics) performed in Lyon, France and/or Quindio Colombia Reference Laboratories [10, 25]. We also tested an additional 25 participants in the National Collaborative Congenital Toxoplasmosis Study (NCCCTS) and our other studies during this time frame who wanted to participate.

Providers joined the study as collaborators following an Obstetrics Department Grand Rounds and Obstetrics Sectional Educational informational meeting for those who missed the Grand Rounds. Both informational meetings were presented by RMc. Providers were provided the same educational pamphlet that their patients also received. All had the opportunity to ask questions of RMc. As described above under “Patient Recruitment”, providers then mentioned the study to their patients. At the initial and subsequent visits the medical student (JL), Maternal Fetal Medicine Nurse (KL), or PI (RMc) obtained the samples after coordinating with the patient and practitioner at the time of a subsequent monthly obstetrical visit. Providers were told the results they could discuss with their patients.

Surveys, designed to assess patient satisfaction with the gestational screening program were created to use at the end of the study. Responses were based on a 5-point Likert scale, ranging from “strongly disagree” to “strongly agree.” There was also an opportunity for free response regarding strengths and potential areas of improvement for the screening program. The detail of questions is in a figure in the results section. Surveys were provided by the research nurse or others working in the study to the study participants at the 6-week postpartum visit or shortly before this visit. Contact with provision of the brief questionnaire was missed for five study participants at the 6-week postpartum visit. All five were asked and two of those five completed the questionnaire at a later time. Surveys were anonymized, so correlation of survey data to individual respondents was not possible. As part of this intent-to-study, as above, we had enrolled 22 pregnant women, and 19 continued monthly. In the Lyon, France reference laboratory, the 155 samples were tested with Abbott ELISA IgG/IgM. When Abbott Architect (France) IgG/IgM had either an IgG or IgM that was positive, backup testing was performed with VIDAS in Lyon laboratory, and LDBio Western Blot IgG/IgM (IgM performed for three tests at LDBio). In the Quindío, Colombia reference laboratory that uses VIDAS family (VITEK^®^ ImmunoDiagnostic Assay System) as an automated enzyme-linked fluorescent immunoassay (ELFA) test, the last 88 of the 155 samples were tested in parallel.

### Study 4. Collation of Earlier Testing, Bibliographical search, and development of novel paradigm

We collated results of all of our earlier work, both published already [23-27, 29-31, 35] and other separate studies ongoing at present on related topics that are being separately submitted for publication currently [Abraham et al, in submission 2023; 30], and our current work herein. Now that the ICT is CE marked and available in a Europe, to determine whether we had overlooked any other study we were not otherwise aware of, we performed a bibliographical search on Pubmed using terms for evaluations of the *Toxoplasma* ICT IgG-IgM test. This was to make certain that we had included all reported tests in our analysis. Only English literature was reviewed. For evaluations found, full text was retrieved and searched for potential false positive samples. Additionally, results of evaluations presented in congress that were known by the authors including those in submission to other journals were added to the totals in this analysis. Earlier and ongoing studies and reports were arranged chronologically and the total collated data are reported herein.

### Summary diagram of Novel paradigm the work presented herein develops

Difficulties we encountered initially in our clinical trial inspired organizing the algorithm we created and show graphically in **Figures S2.1** and **3** to prevent problems like those we had to address.

### Study/Analysis 5: Time cost analysis

We found a number of approaches including reference laboratory tests with varying costs, ease of use, and considered whether they meet WHO ASSURED criteria. Advantages and disadvantages of those approaches are summarized in tabular format including a time cost analysis.

### Study 6: Evaluation of instructional materials for ICT with whole blood at point of care in limit of detection/quality of instructions (QI) study in accordance with FDA/CLIA guidelines

The following study was performed to determine the precision of the ICT with samples at the limit of detection and whether never experienced testers could read, understand, perform and interpret instructional material for use of the ICT with whole blood. Samples were prepared in accordance with FDA/CLIA requirements and guidance for instructional material for CLIA waiver for a point of care test (Recommendations for Clinical Laboratory Improvement Amendments of 1988 [CLIA] Waiver Applications for Manufacturers of *In Vitro* Diagnostic Devices ([version of January 30, 2008 - in force and updated in 2020]). The following limits of detection, “Quick Instructions” (“QI”) study was then performed as follows: Nine testers were identified. Testers were three practicing physicians, three nurse/nurse practitioners, two medical students and one medical resident. They were not experienced with this type of ICT using whole blood or this cassette. None worked as a certified laboratory technician. They were selected to reflect categories of potential users of this test who were unfamiliar with and unskilled with this test. Each tester took the University of Chicago blood-borne pathogens and universal precautions training to work with whole blood, and their competence in understanding and using this material currently was formally documented for the study, as was IRB- required. The ability of three groups of testers with different clinical roles to read the instructions, to perform the test in accordance with the instruction, and to distinguish negative and positive results at the pre-established limits of detection were evaluated. Additional methodologic details are in the Supplement.

### Study 7. Detection of acute infection and seronegatvity in Quindio, Colombia by using ICT

Sera from 22 patients who had recently acquired their *T.gondii* infection in Quindio Colombia and 12 patients who were seronegative had sera that were tested with Vidas ELIFA IgG, IgM and with ICT.

### Study 8. Additional NCCCTS patients and their families had testing with ICT while at follow up visits in Chicago to add data determine antibody present for many years is still detectable by ICT

Between March and December 2018 20 participants in the NCCCTS whose serologic status was known from earlier reference laboratory testing and family members traveled to Chicago. Number of tests were 20 seropositive and 8 family members or other controls for separate studies such as multimodal neuroimaging studies were found to be seronegative but did not have other reference laboratory testing. Time from acquisition of infection was noted, in a similar approach to earlier studies of Begeman and Lykins.

### Study 9. Use of ICT for Epidemiologic study in Cincinnati

Sera and demographic data from a maternal-infant cohort in Cincinnati were available for 265 women; 264 had data on the variables of interest. Variables of interest included residential address (longitude and latitude), age, education, race, income and pet ownership as part of the original cohort study. Sera were tested with ICT and if positive then were tested with IgM and IgG western blots at LDBio. A logistic regression model on the results for the 264 samples was used to estimate the ICT *Toxoplasma* infection positive status by including independent variables such as, maternal age, marital status, Neighborhood Deprivation Score (a higher value means more deprived and missing values are extrapolated from 5 nearest neighbors), latitude, longitude, race, i.e., White or not, and an interaction term between maternal age and Marital status.

### Study 10. Evaluation of lateral chromatography test AdBio that detects anti-*T. gondii* IgM and IgG separately

To determine whether a USA made immunochromatography test (called ADBio) woul function as well as the ICT or whether samples from Colombia that had very high performance with ICT would function as well with a different USA made test, an additional set of known positive IgM positive or IgM negative samples was tested. The tests that were used were the VIDAS (Quindío, Colombia) test and another commercially available but not FDA cleared or CLIA waived test that detects *Toxoplasma* specific IgM in the Colombian Reference laboratory. A total of 147 serum samples were included, selected from the biobank of past studies at the University of Quindío in Armenia, Quindío, Colombia. All samples were previously tested using the reference test VIDAS (VIDAS Toxo-IgG Avidity kit; bioMérieux, Marcy l’Etoile, France). Samples were divided into the following three groups as defined by VIDAS testing: (1) IgG negative and IgM negative (n=65), (2) IgG positive and IgM negative (n=55), and (3) IgG positive and IgM positive (n=27).

### Analysis 11 : Representative Case Summaries illustrative of practical clinical problems where solutions are needed and potential utility of ICT

Representative case vignettes with concepts they illustrate were collected and were summarized to illustrate impact and need of this paradigm and its historical context (as illustrate in “**Studies 2a,b, and 10”**). These brief case summaries are from The National Collaborative Chicago-Based Congenital Toxoplasmosis Study and Consultations to the Toxoplasmosis Center and Toxoplasmosis Research Institute. They illustrate representative, frequent clinical problems incurred from false positive IgM tests. Representative examples of benefit and novel utility of ICT in addressing this problem are also presented. A case summary also presents use of ICT for early detection confirming pre-conception infection when sequential samples obtained in the context of *in vitro* fertilization (IVF) were available. Commentary about screening programs and their absence in the USA further place our findings in an historical perspective in the Supplemental.

#### Ethics

The ongoing UCMC study, under the name “Prevention of CT: Feasibility of prenatal screening using point-of-care tests,” is conducted with ethical standards for human experimentation established in the Declaration of Helsinki. Research received approval from the University of Chicago Institutional Review Board (University of Chicago IRB Protocols 20-0442, 19-0505, 21-1446, 8793, 8794, 8795, 8796, 8797, 8798, 16708A and met the standards of the Health Insurance Portability and Accountability Act. Results were or will be reviewed by/discussed with the Data Safety Monitoring Board. Informed consents were obtained from subjects in accordance with the University of Chicago Institutional Review Board and the National Institutes of Health guidelines. No subjects are under age 18 years. All participants provided informed, written consent prior to their study participation. All studies were performed in accordance with the Declaration of Helsinki.

Samples from the Lyon Reference laboratory of the University hospital were anonymized in this analysis. Testing in Colombia was performed in accordance with local Ethics Committees approvals and guidelines. Stored sera from Cincinnati was approved by the IRB for future testing for a wide range of pathogens.

## RESULTS

A more detailed version of the Results including Tabulated primary data and approaches in Figures are in the Supplement.

### Feasibility clinical trial study performed exactly as the test would be used in practice, 2020 to 2021 demonstrates feasibility, identifies false positive predicate test results and develops new paradigm to help to obviate that problem, Study 1

**Figure 2.**
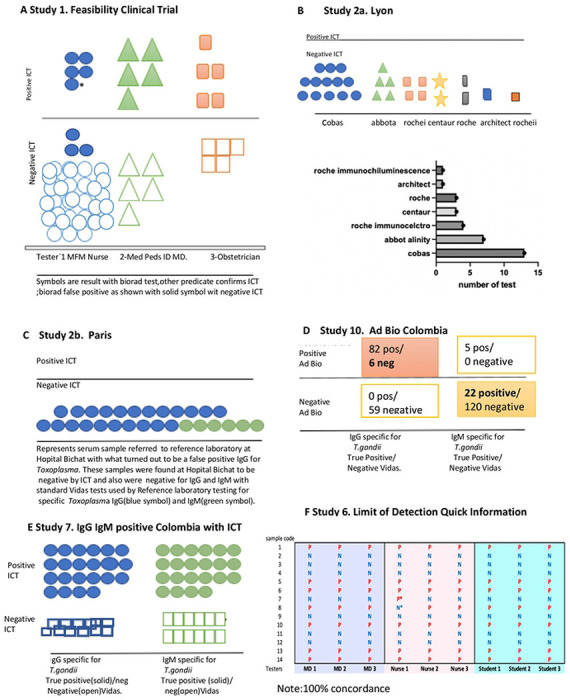
High performance of ICT **A. Clinical Feasibility Trial.** Solid symbols represent positive predicate test, open circles represent negative predicate test.* False positive IgM predicate test for seropositive person. **B. ICT is negative with 32 false positive standard tests in Lyon. C. ICT is negative with false positive standard tests in Paris,** Blue IgG, green IgM. **D. ADBio, a USA test, suffers from false negative and positive IgM results. E. ICT detects acute infections with positive IgG and IgM in Colombia. F. Quick instructions** are able to teach 9 testers, 3 physicians, 3 medical students and 3 nurses to use test accurately with 7 samples that are negative and 7 samples at the limits of detection. Detailed methods, results and figures are in the Supplement for Figure 2 A. **Figure S1A-E, Table S1; B. Table S2; C. Table 3; D. Table S8; E. Table S7; F. Figure S4**.

Individual results in the ICT and the predicate test in this ongoing clinical trial study are in Figure 2A and **Supplemental Table S1**. Between August 2020 and December, 2021 we performed a prospective clinical trial in which 43 seronegative and 15 seropositive persons were tested with the ICT using whole blood and sera. The 58 sera were also tested with the predicate test used by The University of Chicago Medicine Clinical Serology laboratory, the Biorad assay. Results of all readers of the ICT were uniformly concordant. Any positive results were tested in Reference Laboratories.

**Figure 3.**
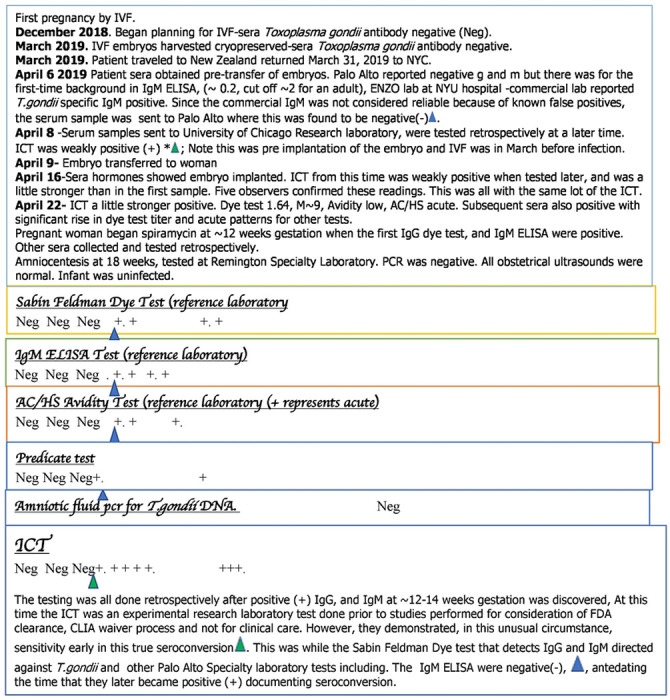
Representative example of ICT detecting very early seroconversion using sera originally stored for another purpose tested retrospectively, showing contrast with false positives Blue triangle marks tests at time of seroconversion detected by ICT marked with green arrow. + represents positive result. There are a few examples of ICT positive with IgM only early and others G and M only early in seroconversion.

**Figure 4.**
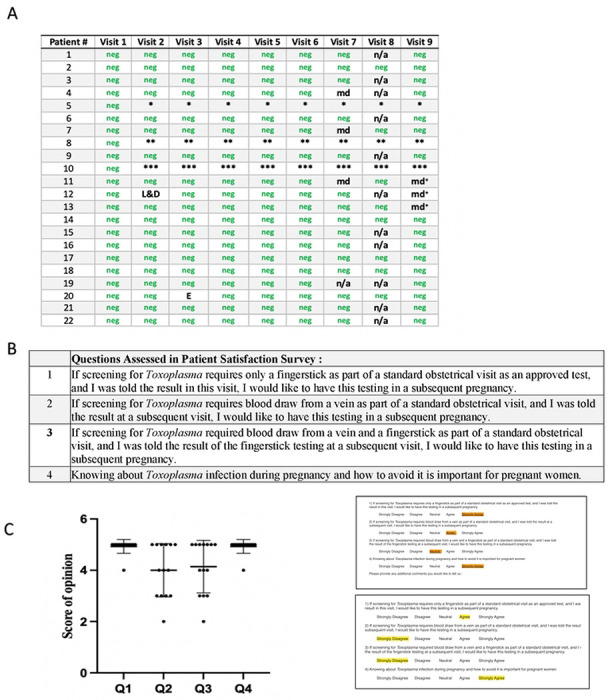
Feasibility and acceptability of monthly screening in U.S. Academic practice Abbreviations in A.:**“neg”** indicates negative results in tests. Note congruence of ICT and Reference Laboratory test conclusions. * Elective termination secondary to fetal anomalies, ** Spontaneous abortion, *** Elected to leave the study after first test time due to traditional beliefs regarding having blood drawn “md” indicates a missed appointment, as patient did not attend her regularly scheduled appointment. Came at alternative time and we did not connect for testing and/or survey. “n/a” indicates not available “L&D” indicates that the patient’s monthly test was missed due to concern for premature labor, which resulted in a visit to the emergency department and then labor and delivery “E” indicates an equivocal result, in which a barely visible band appeared which was not reproducible upon photographing the test. Per manufacturer instructions, this test was interpreted as negative. +Survey sent later, not included on graph as was not at initially planned time of six-week postpartum visit^a^ **A. Times and results of monthly screening for each participant. B. Survey questions and Likert scale.** n the survey, number responses were considered as follows: strongly agree was 5, 4 was agree, 3 somewhat agree, 2 was somewhat disagree, I was strongly disagree **C. Results of satisfaction survey for 14 participants.** Each respondent’s answer is represented by the circles in the figure. The mean is indicated by the horizontal line, with error bars indicating standard deviation. In response to questions 1 and 4, 13/14 respondents indicated that they “strongly agree” that they would pursue testing for *T. gondii* in future pregnancies with POC testing, and that knowledge of *T. gondii* is important for pregnant women. Results were more mixed if testing required venipuncture, as indicated by the responses to questions 2 and 3, but most agreed that the test was important, would have it again in a subsequent pregnancy, and would recommend this to family and friends. It was noteworthy that other family members such as fathers of the fetus asked to be tested, relevant to the possibility of retinal disease. Right panel showed results after the time of the 6 week post partum visit. We did not conduct a formal survey at the time; however, we began with two providers, and other providers in the practice asked to join with their patients. Providers continued in the further analysis of the ICT as it moved toward clearance and waiver.

Within testing of the initial 6 pregnant participants, we encountered false positive results for two participants in the IgM predicate test and three others later revealing a false positivity rate of 10%. Finding these frequent false positive predicate test results was disturbing for patient participants, providers, and investigators. Correcting the erroneous predicate test data with follow up gold-standard testing was time consuming, costly, and results in delays in care. Although the resource Reference laboratories in the USA and France provide one excellent solution to the problem of false positive results, that is of high quality results, the delays, cost and inconvenience were substantial difficulties. This occurred while we as investigators could promptly see the negative ICT result in whole blood and sera testing. We knew the results in the context of our earlier data with very high performance, sensitivity, and specificity of the ICT. Our earlier work had demonstrated negative results were accurate with the ICT for erroneous predicate test results considered as positive when samples were referred to the USA reference laboratory. This occurred when 33 patients with 60 sera were tested (Tables 1 to 3). We recognized that if we could not solve this problem of false positive predicate tests, this would have de-railed the research study and its longer-term goal of proper testing in systematic gestational screening programs for the USA and as a model for other countries. For the USA, along with their present high costs in the USA, false positive tests also would substantially harmfully influence the use of screening programs (Figure 3, 4, **S2.1**, Tables 4, 5, **S6**).

### New paradigm elucidated by this experience

Thus, as we tested the whole blood and sera with the ICT in parallel with testing the sera with the Biorad test, we developed an easy, inexpensive paradigm shifting approach to solve this problem of false positive tests for the USA. This paradigm (Tables 4, 5, **Figures S 2.1, S 3**) showed the ICT could help to eliminate the problem of false positives both in the clinic and the clinical laboratory. This paradigm was to have a method for diagnosis with a test that meets WHO ASSURED criteria available promptly at the time the test was performed and to have a first backup of positive results in serum rapidly in the local laboratory. Our experience shown demonstrates that this ICT performs properly in clinical practice and field studies. We noted that it could be used correctly by previously untrained observers, meeting WHO ASSURED criteria. We found that this also could help to obviate the difficulties caused when a commercial predicate test has false positive result. This was while introducing a novel test that could be low cost and easy to use in the clinic. It became clear from the experience and data presented that this novel test and paradigm could be a useful new method for the clinical laboratory to identify true positives rapidly for this emergent problem/disease. This could help to determine whether there was need for further screening. It could help to clarify whether there was need for emergent care with life, sight, cognition saving medicine should be initiated promptly while waiting for backup reference laboratory confirmation of a true positive test.

### Additional testing of erroneous false positive local predicate tests with ICT and gold-standard testing in reference laboratories demonstrates utility of the novel paradigm with ICT, Study 2

Then, Lyon and Paris Reference laboratories’ identified erroneous false positive results reported for samples referred for testing from local private laboratories that used commercial tests. Thirty-two samples that were referred to the Lyon reference laboratory from September 2021 to February 2022 from private laboratories because of the detection of isolated IgM in the course of monthly prenatal retesting, which the main system used in France in this context (Figure 2B, **Table S2**): The tests that had been used included Cobas Roche (n = 21), Abbott (Architect n = 1 or Alinity n=7), Siemens (n = 3) (Figure 2B, **Table S2**). None of the additional 32 samples gave positive results with ICT or in the reference laboratory with Abbott Architect despite the erroneous reports of positive IgM results (**Table S2**). Further, in Lyon France, none of the 32 false positive IgM tests with the predicate local laboratory tests used had false positive test results with the ICT or gold standard Western Blot (**Table S2**). This was using the same referred serum that was tested and reported to be positive from the local private laboratory. These results are included in Tables 3 **and S2, and** Figure 2.

### Placing US ICT in the context of other ongoing studies, including Study 3 and previously published studies, demonstrates high performance, Analysis 4

Data summarized in Tables 1 to 3 place the results in the Clinical Trial and monthly screening acceptance studies (**Study 3**), in the context of other ongoing studies and our earlier published work. Table 3 addresses details of studies in the USA and elsewhere. Tables 1 to 3 also collate and address studies of false positive IgMs referred to reference laboratories in the USA and France. Table 3 collates all these studies including those of other countries as a summary of all available results. Sensitivity, specificity, confidence intervals and details of studies are in Tables 1 to 3. Performance of ICT is high, sensitivity≤ 98.5%, specificity ≤98.9% (serum and/or blood).

Additional testing of a set of samples with Architect, Vidas and other tests was performed in an additional matrix analysis pertinent to consideration of false positive test results. Back up testing of another set of sera from a monthly screening and acceptability of the monthly screening program was performed in the Lyon France and Quindio Armenia Colombia Reference laboratories (**Table S6**). In these reference laboratory settings as well as in the Paris Hôpital Bichat reference laboratory [30] false positive test results were less problematic (Figure 2 C, **Table S6**) than in the clinical trial. There were no false positives in the Quindio laboratory Vidas testing and one patient with multiple consecutive IgG false positives in the Lyon Architect tests.

### Bibliographical search confirms high performance and that data analysis herein includes all published studies

As the ICT is now commercially available following CE Mark approval in Europe, we also used a bibliographic search to attempt to identify results with which we might have been unfamiliar or with inferior performance of the ICT. Table 3 details all studies performed to date: Number of persons (N), Sensitivity (Se)/ Specificity (Sp), country of samples, N of false positive IgM, ICT results on false positive IgM and confidence intervals, testing for serum and whole blood are in this Table. As the ICT is now commercially available following CE mark approval in Europe we also used a bibliographic search. To date all studies have involved the authors of this manuscript. There were no additional studies identified that have been reported to date. A total of 4606 sera, 1401 positive and 3205 negative, and 1876 whole blood tests, 728 positive and 1148 negative tests have been performed, including all published, ongoing studies and those herein with high sensitivity and specificity (Table 3).

### Overall performance of ICT with NRL tests false positive IgM also is high, Study 2 a and b

Overall, including our own results herein, we found 137 samples with false positive IgM in at least one NRL technique also tested with ICT, among which 132 were found negative in ICT. The specificity of ICT for false positive IgM was 96.4%. Three samples were from IgG negative pregnant persons in Chicago. Two seropositive persons also had false positive NRL IgM was not found in Reference laboratory IgM tests. In addition 22 false positive IgG results were correctly identified at Bichat-Claude Bernard Hôpital, Laboratory of Parasitologie, Paris, France [30](Figure 2C, Table 3) and 5 false positive IgG identified in the predicate Abbot Architect in the University of Chicagomedicine samples tested in Institut des agents infectieux, Hôpital de la Croix-Rousse, Lyon, France (Table 3).

### Time, cost, comparing tests demonstrates time/cost savings and aids in eliminating delays, Study 5

An analysis of relative time and cost is in Tables 5. The ICT is substantially time and cost saving,

### Representative case summaries illustrate problems in care that false positive tests can cause and utility in identifying seroconversion in infection acquired prior to conception, called study 4b and “analysis 11” in methods)

**Table S8** discussed in **Supplement Commentary** has brief summaries of some consequences of false positive and negative results in ongoing cases in USA clinical practices. These provide further evidence of problems that false positive test results cause.

Figure 3 **(Study 4b)** shows utility in identifying infection prior to conception. The ICT detected seroconversion a day earlier than the Sabin Feldman Dye test and IgM ELISA in the Reference laboratory. There are a number of examples of patients who developed M alone then M and G [26]. In the Mahinc et al study [26, Table 3] there were 50 serum samples from 24 women for whom there were 17 samples with IgM only and 33 samples with IgM and IgG; ICT was positive for all samples except one that had a borderline IgM ISAGA of 5 for a patient who later was found to have acute *Toxoplasma* infection. There were also another 144 acutely infected persons identified in the USA, France, Morocco and Colombia all identified as positive with the ICT [24, 25, 27, 29, 30] Table 3. It was unusual, however, to watch seroconversion with as much precision in narrow time intervals so early in infection as shown in Figure 3.

Figure 3, **compared with Figures S7 and 8** contrast current status and consequences of CT at earlier times and continuing to present in France and in the USA. This illustrates that the ICT and gold standard back up testing can solve a substantial health care problem. This is both in a historical context and at present, with potential spillover benefit for care for pregnant women and their families.

### Testing ability of written instructional materials to facilitate clinical use of the ICT by healthcare practitioners not skilled in using the ICT in a limit of detection quick information study per FDA and CLIA instructions demonstrates high performance, Study 6

Moving toward implementation, ability of written instructional material to be used in clinical practice with samples at limits of detection for positive whole blood samples and negative whole blood samples was found to have perfect performance. This perfect performance was for all the “blinded” readers and testers performance and reading after they read the Quick Information (QI) materials (Figure 2F, **S4**).

### Feasibility and acceptibility of monthly gestational screening with ICT is demonstrated in Study 3

Early in the work with the ICT in 2017 to 2018 we performed this study to determine whether monthly gestational screening would be feasible in a research study setting (Figure 4, **Table S6**). Results of the testing did not enter standard medical records or the EPIC system at that time and the testing took place earlier than study 1 but was completed after that study. This study was initiated before the clinical trial and led to the initial meeting with the FDA when a program officer from the Thrasher Foundation emphasized the importance of FDA clearance for the test to be useful to help patients in the USA. We also asked participants at its completion whether participants felt it was important and comfortable to have knowledge about *Toxoplasma* in pregnancy and whether they would want serologic testing and/or the finger stick point of care test in subsequent pregnancies if it were approved in the USA. The intent was to determine whether screening might be acceptable in standard academic obstetrical USA practice: Some parts of the study, e.g., the questionnaire and additional backup testing were performed in 2020. Results for the initial tests for the participants’ visits were included in the earlier 2018 publication [23]. Thus, numbers included for this study in the cumulative total of tests were subtracted from tests shown in Figure 4.

Patients were all identified at between 8-12 weeks gestation. Patients had a median age of 31 years (range: 24-40 years). Seven of the 22 participants were nulliparous, while the remainder had been pregnant once or twice before. None reported having been tested for *T. gondii* infection in the past. Participants were enrolled in the study between September and November, 2017. The study initially concluded in September, 2018 with the birth of the last participant’s child. Because five mothers were missed by our study group at their 6-week postpartum visit, an anonymized questionnaire was provided in 2022 for those participants. This was considered separately in our analyses. Patients were tested at monthly intervals after their initial enrollment and tested until their 6-week postpartum visit. A small subset of patients (3/22) were withdrawn from the study: One individual underwent elective termination due to fetal anomalies. One participant suffered a spontaneous abortion. The third patient chose to withdraw from the study due to traditional beliefs about dangers associated with venipuncture. No patients (0/22) had evidence of prior infection with *T. gondii* upon their initial testing with the whole blood POC test, and none seroconverted during gestation. One participant had a faint band suggesting the possibility of a positive test on one test, but this was only visible to the naked eye and could not be independently confirmed with photography. Per manufacturer instructions, this test was interpreted as negative. There was 100% concordance between testing interpretations of the POC test and confirmatory testing, including the ARCHITECT/Vidas/Western blot systems and the serum-based POC test variant, commercially available and now CE mark approved in France. The course of gestational screening for each participant is presented in Figure 4, **Table S6**.

Initial response of patients and their families to screening was noted. No patient declined and responses were enthusiastic from patients. There were even requests from patient participants about whether other pregnant and non-pregnant family members and friends could join. For example, even some fathers asked to be tested to know their own serologic status and if they might be at risk of retinal disease if infected. At the end of the consecutive screening tests, we administered the patient preferences survey to an available subset of the cohort (14 in total) at their 6-week postnatal visit. Those participating women who were missed completed the questionnaire in 2022. The POC testing and screening for acquisition of *T. gondii* in gestation was well received by all participants. There was not a formal questionnaire for providers. Rather, level of interest and enthusiasm was reflected by the following: All providers remained involved in the study with their patients. Additional providers in the practice noting the ongoing study with the initial providers asked to join. Those still practicing at the University of Chicago at the later time did continue to collaborate in the subsequent clinical trial study presented herein. These objective measures documented continued involvement rather than a formal survey. All providers found the finger-stick testing and monthly screening a constructive addition to their practice. The rapidity of obtaining the results was viewed positively.

### ICT detects early seroconversion and distinguishes additional seropositive and seronegative samples in USA, France and Colombia, Studies 4b, 7 and 8

We noted, as shown in Figure 3, the ability of the ICT to detect very early seroconversion in a study of sera obtained at narrow intervals to monitor hormone levels during *in vitro* fertilization (IVF), that happened to occur during very early seroconversion. This was a USA patient whose *in vitro* fertilization had occurred 6 months prior to implantation of their embryo at a time that she was seronegative. In the interval between *in vitro* fertilization and implantation she had traveled to New Zealand where she likely acquired *T.gondii* infection in the weeks before implantation as shown in Figure 3.

Our earlier studies (Tables 1, 2, 3) have all identified perfect performance in detecting sera from those with acute infection in the USA. It was, therefore, of interest to determine whether the same would be found in sera from patients with acute infection with the genetically different parasites found in Colombia. Figure 2E, **Table S7** shows perfect ability, sensitivity, and specificity of the ICT to also identify acute infection (IgG, IgM) in Colombia (n=22) and those who are seronegative (N=12), (p<0.0001)(sensitivity 100% and specificity 100%). This brought the total to 144, as above, Further, serologic status was correctly identified for additional NCCCTS participants tested with finger-stick whole blood and serum between March and December 2018 herein (N=20 positive chronically infected persons [times after infection years were known to be greater than 17 years for all except 3 persons, and 5 negative). As we found perfect correlation of testing of whole blood obtained by fingerstick and serum testing in the United States, herein, and almost perfect correlation in Morocco this ICT using whole blood accurately distinguishes seronegative and seropositive status as occurs in seroconversion. Indeed, in the study in which we tested whole blood (that contained serum that originally had 38 UI/ml of IgG and 63.89 ratio for IgM according to Roche *Toxoplasma* kits, diluted 1:89 in whole blood from a seronegative donor) at the limits of detection in the “QI study”, there was high accuracy in distinguishing positive and negative samples (Figures 2F, **S4**). There were N=63 negative and 63 positive, making a total of 126 tests of samples performed. There was 100% accuracy both with the cassette and with photographs read by the tester and two additional readers. All readings were congruent and consistent. All were blinded for 9 testers with 3 testers in each of three settings (physician office, nurse health care setting, and laboratory conference room], and with testers differing professional backgrounds (3 nursing, 3 medicine in training, 3 licensed physicians in practice previously unskilled in use of such a test) (Figure 2F, **detail in Figure S4**),

### Use of ICT for Cincinnati epidemiology study between 2017 and 2019 demonstrates that ICT is an efficient way to perform such studies and that prevalence is low in Cincinnati, Study 9

**Figure 5.**
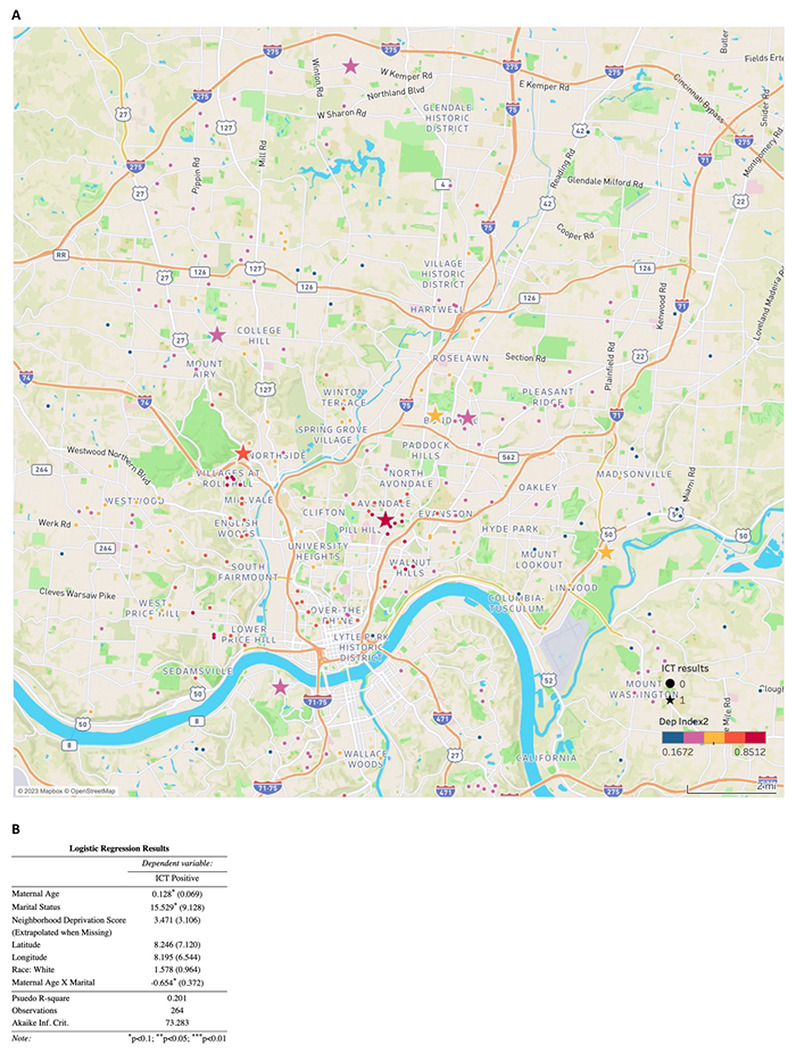
Location of seropositive persons in Cincinnati and associated demographic factors such as socioeconomic status, maximum educational level achieved, pet ownership, and ethnicity Sera were collected between 2017 and 2019.The low prevalence of seropositivity did not allow testing for clustering by any known risk factors for infection, including proximity to watersheds associated with sewage water run-off. None of the sociodemographic parameters, neighborhood deprivation, nor residential latitude and longitude measures achieved statistical significance. The measures of neighborhood deprivation are indicated by the color of the symbols.

Of the 265 mothers tested, 8 (3%) had a positive IgG for *Toxoplasma* infection. None of these had a positive IgM. Variables of interest were available for 264 of the mothers including residential address (longitude and latitude), age, education, race, income and pet ownership as part of the original cohort study. There were no significant associations of testing positive for *Toxoplasma* infection and any of these variables (Figure 5).

Results with ICT AdBio test (Onsite POC) that detects anti-*T. gondii* IgM and IgG separatelyhas both substantial false negatives and false positive IgG (9%) and false negative IgM(18.5 % true positives detected), Study 10.

We hoped that a test developed in the USA called ADBiothat is purported to distinguish IgG and IgM specific for Toxoplasma might be useful in a field setting. This test had a high proportion of False negative and substantial number of False positive results (Figure 2 D, **Table S8**). Please also see detail in Supplemental.

## DISCUSSION (Also Expanded Introduction and Discussion are included as the Commentary in the Supplemental)

The results above demonstrate that the ICT has proven effective at identifying sera and whole blood samples of USA and non-USA patients with known *T. gondii* infection. It detects seroconversion early in infection. It is also was effective at identifying the false positive test results for *T.gondii* specific IgM of other, currently FDA cleared tests of sera when no *T. gondii* specific IgG is present. It was well-accepted in a monthly screening program that was shown to be feasible in a USA academic obstetrical practice. It also functioned with high precision while meeting WHO ASSURED criteria even in whole blood samples at the limit of detection of specific anti-*Toxoplasma* antibody. It was found to be straightforward for physicians, nurses and medical students and a medical resident to easily learn to use the ICT and accurately interpret the ICT results using the Quick Information in simple written instructions (Figures 2F, **S4**).

Up through and including the current stages of the clinical feasibility trial at the University of Chicago Medical Center, diagnostic sensitivity has exceeded 99% and specificity has stayed at 100% with all samples of U.S. patients. In addition, across several of these studies, this ICT has outperformed other screening tests. Herein, out of 99 IgM false positive sample results, across multiple consecutive different USA and French sets of data recently there have not been false positives or false negatives. In addition, in two countries (the USA and Morocco [29]), the ICT has not had false positive or borderline bands when testing the serum and/or whole blood. While it was already known that this test could perform accurately (Tables 1–3, **S1-7**), this present work also has evaluated the ability of ICT to correct the errors of other carefully tested, commercially available screening assays [27] using prospectively and retrospectively collected sera in the USA, France and Morocco. The high specificity is a particular strength for the ICT IgG-IgM device, especially when compared to other currently available commercial tests for anti *Toxoplasma* IgM.

The data from Houze et al (ECMID and manuscript in preparation [30] and Mahinc et al [26] increases the number up to 137 of such false positives IgM studied with the ICT. Mahinc et al also studied 23 false positive Architect and/or Biorad Platelia IgM [26]. In the Mahinc study [26], false positive IgM in the Biorad test were obviated by ICT testing 21/23 of the time. In Tunisia [31], recent results were similar adding additional data but with a higher proportion of false positives [31]. Ten of 13 false positives were negative in the ICT. Although there were no ICT false positives in these data sets in the US, the occasional false positives (5 of 36) in the work earlier in Marseilles and Tunisia emphasize the importance of confirmatory testing of positive results. The high quality performance of some of the Reference tests emphasize that some tests seem to perform better than others when used in Reference laboratories (Figure 2E, **Table S6, S7**).

Our studies, along with the earlier experience in the Palo Alto reference laboratory and collated recent results, demonstrate practical problems in the US with potential serious consequences for patient care [35] where the ICT can be helpful in a patient’s management. This has been confirmed in France making a total of 132 of 137 for IgM) and 27 of 27 times for IgG times that a false positive result could be corrected. False negatives are uncommon but would be detected by repeat testing in gestational screening programs. Any positive ICT during gestation would have confirmatory testing to differentiate IgG and IgM. The occasional false positives would be detected by back up testing in the reference laboratory in the USA or use of multiple tests including the Western blot in France. Reference laboratory gold-standard testing and certain commercially available test reagents have higher performance than testing in local laboratories as shown in Figures 2B, 2C, 2E, Table 3, **S1, 6**. The ICT has high precision with samples at the limit of detection. That the test is easy for medical students, a medical resident, practicing board certified physicians, nurse/nurse practitioners without familiarity with the ICT to perform and interpret (Figure 2F, **S4**) is congruent with a recent experience with 30 practitioners in Armenia, Colombia [47]. This experience was with patients infected with genetically distinctive Colombian parasites [47]. Acceptability in a Colombian patient and obstetrical practitioner group was high [47], similar to acceptability in our USA experience presented herein.

Colombian sera also were tested in Colombia [47-50] with a different lateral chromatography test made in the USA called the ADBio. This test differentiates IgG and IgM and has a USA sale price more than ten times that expected to be applied for the ICT. Unfortunately, the performance of the USA manufactured ADBio test was problematic (Figure 2D, **Table S8A-C**) when compared in the Quindio Reference laboratory with Vidas IgG and IgM reference tests [47-50]. This is similar to our earlier results with this test with French (unpublished) and USA [27] sera. For the Colombian sera specifically, there was a marked difference of the ICT and combined detection of IgG and IgM antibodies: The AdBio test resulted in lower sensitivity for IgM in stored samples from a biobank. ICT combined simultaneous detection of IgG and IgM can improve sensitivity for IgM because most of the IgM sera used for sensitivity analysis already have IgG [26, 27, 30} and the mechanism of the test with antigen coating the black bead reacting with both quadra/pentavalent IgM and bivalent IgG which react with the antigen placed in the line on the nitrocellulose. This combined detection of different isotypes also contributes to better specificity. The lysate antigen used in the ICT contains many proteins. The Western blot can accurately discriminate between and recognize IgG and IgM specific for T.gondii, as can the combination of other tests such as the Sabin Feldman Dye test which also detects IgG and IgM and the double sandwhich IgM ELISA or the IgM ISAGA. The IgM ISAGA is more sensitive and thus preferable for use for infants.

In the context of clinical protocols for prenatal Toxoplasma screening, the ICT insures that far fewer “false alarms” are generated and that less time and resources are spent on confirmatory testing for a pregnant woman who shows an isolated positive Toxoplasma IgM test. Risk that such sample may be a false negative IgM from the ICT test is very low, but cannot be excluded. To avoid any risk the patient should be retested for IgG and IgM 2-3 weeks later to ensure that IgG did not appear. This happens as part of a systematic gestational monthly screening program (Figure 4, **Table S6**). It should be emphasized again that POC tests for anti-Toxoplasma IgG and IgM, such as the ICT, are merely a first step toward diagnosis, given that IgM antibodies can persist for up to several years after acute infection. For any woman who receives a false positive IgM test result, the next step of an evaluation with other tests can involve weeks of waiting for a blood sample to be tested using technology that runs at much higher costs than the point of care test [1, 2, 6, 7, 27, 33, 35].

A potential limitation of the ICT might have been the lack of utility of the ICT using saliva (Peyron, unpublished). There is a nanogold Nirmidas test that was used with saliva, serum, and whole blood finding high sensitivity and specificity and dye test precision for the detection of IgG and IgM [37]. We had suggested earlier this might be an ideal test to use before conception. Although finger stick for glucose is standard, easy, and familiar in obstetrical practices, obtaining saliva may be viewed as less difficult than whole blood. Thus, some view saliva could be a potential advantage. However, the nanogold has required transport, associated delays to reach a clinical laboratory, and electricity and a sophisticated machine for testing. Recently manufacture of this nanogold test was discontinued. Nirmidas has also used a gold bead ICT for SARS CoVi2 but nothing like this has been produced for *Toxoplasma* to date. The diagnosis and management of *Toxoplasma* infection best involves knowledgeable health care provider input urgently making the advantage of home testing saliva less.

Testing before conception to identify seropositive persons and then testing regularly monthly through pregnancy for those who are IgG seronegative initially would be ideal as it helps to obviate problems of anxiety provoking delays that can result in irreversible fetal damage, as well as false positive test results. Such damage in congenital toxoplasmosis, as well as in ocular toxoplasmosis can occur in very short times of less than a week, making diagnosis and initiation of treatment urgent and emergent. Minimizing the likelihood of false positive IgM while maintaining maximum sensitivity is a top priority for any point of care test candidate.

The ICT also should be very useful in clinical laboratories testing with sera with a potential false positive IgM result without IgG as described herein. It could function as a second-line test to confirm or find IgM specific for *T. gondii* is not present before sending the sample to a reference center, while continuing to follow the patient while awaiting Reference laboratory results. This is a major advance as this will save time and reduce the need for gold standard tests. It can help reduce concern for patients and physicians.

When the ICT test is used initially with whole blood the only predicate test for confirmation needed will be if the whole blood test is positive. ICT not only helped to obviate the problems with false positives but also can result in detection of true positives and very early seroconversion as described herein and also recently acquired infections described elsewhere [9, 23, 24, 26, 27, 30]. We placed this work in the context of ongoing problems for healthcare (**Commentary Figures S7, S 8**) and potential for direct and spillover benefit for the care of pregnant women and their families (**Commentary Table S9**, [14]). We also placed these studies 1 to 12 herein in a historical context building parts of a toolbox working toward a role of screening using WHO ASSURED criteria compatible tests in the elimination of congenital toxoplasmosis (**Commentary Figure S7**). There also are a variety of other clinical and epidemiologic circumstances where knowing *T.gondii* serologic antibody status can be of considerable clinical and public health utility and benefit [1, 2, 6, 7, 27, 32-9, 46-50]. Very high-quality, low-cost screening tests such as the ICT can improve infectious diseases care in gestation, help to eliminate perinatal infections with considerable spill over benefit for health care for pregnant women and in other clinical and research settings.

Congenital toxoplasmosis is a treatable and preventable disease, and physicians and other obstetrical providers now have the tools, in-hand, to improve outcomes and reduce patient and familial suffering. This screening, the standard of care in other countries, is now increasingly feasible in countries like the United States, where the primary argument against screening has been its economic burden. In the development of this test and other high-functioning point-of-care tests, there is potential for transformation in the provision of obstetrical care to improve maternal-child health. These benefits are amplified in subpopulation demographics in the USA[28] and regions of the world where the burden of disease is even higher. Examples of this occur in the Lancaster Amish population in the USA [8, 12, 32, 46], parts of Florida, are likely in other US subpopulations [28], and occur in Central [36] and South America [47], and parts of Africa [29].

Use of the ICT for the Cincinnati maternal cohort study found ICT to be efficient (**Study 9**). Due to the small proportion who were seropositive, we were unable to test for any clustering by known risk factors for exposure: none of the individual socio-economic or location factors in a regression analysis achieved statistical significance. Future analyses with a larger overall sample size will be needed to evaluate risk factors in this population. The reasons for the relatively low prevalence in Cincinnati in this cohort remain to be discovered. We have cared for children with congenital toxoplasmosis in Cincinnati, thus, even with the low prevalence found, it is likely still that gestational screening would be worthwhile.

Our recent study in Colombia also demonstrated high acceptability of a single use of the POC on a large scale of 783 women and 30 providers [47]. Although *Toxoplasma* infections occur in all demographics it was a particular problem in those who had lower education and socioeconomic status [31, 47]. To understand risk factors during gestation and develop programs to prevent such infection will require monthly screening in areas of high to low prevalence.

The implementation of this study in the clinical trial and the QI limit of detection study demonstrated that it should be easy to introduce this test into obstetrical or other practice with little time or inconvenience. For example, when patients are evaluated for vital signs, blood pressure, glucose including by fingerstick, by medical assistant or nurse, the cassette can also be brought to obstetrician or other health care practitioner for additional reading and entry into the medical record. Photography using an I phone for documentation could easily be included into the process for additional documentation made available to patient and in the medical record.

In France screening was mandated by law. In Austria those screened received additional health care benefits. In Colombia it was introduced through practice societies. In the USA those in advisory positions recommended that education, easy feasibility, low cost would result in those who would benefit choosing to have testing incorporated in medical practice and USA patient culture at many levels by personal preference. The acceptability study demonstrated that informed patients would want this and obstetricians could use this comfortably and without inconvenience in their practice. It could easily be introduced into family practice and adolescent pediatric care to identify seropositive patients at risk of this most common form of retina disease and loss of sight. Such screening in adolescence could also provide pre-pregnancy testing for young women to allow knowledge of who is seronegative and should be screened during pregnancy. Pre-marital/conception screening as initially occurred in France could also be helpful as families plan to have children. As *Toxoplasma* has been transmitted by organ donation and white blood cell transfusion and by sperm in domestic non-human animals, and can relapse with immune suppression and may be causative for epilepsy, and some neurodegeneration, there are a number of other medical settings where knowledge of *Toxoplasma* serologic status may be useful.

Obstetricians, nurse midwives, family practitioners, obstetrical nurses, and other obstetrical providers are uniquely positioned to intervene to prevent this disease, to improve the health of both mother and child. POC test-based monthly gestational screening of seronegative patients for *T. gondii* infection provides a valuable tool in the obstetric armamentarium to ensure maternal-child wellness. When such tests have undergone appropriate evaluation by the FDA and CLIA, as they have undergone in the CE-mark evaluation and approval in Europe, this testing can enable a paradigm shift in our management of the risks associated with exposure to *T. gondii*.

## Figures and Tables

**Figure 6.**
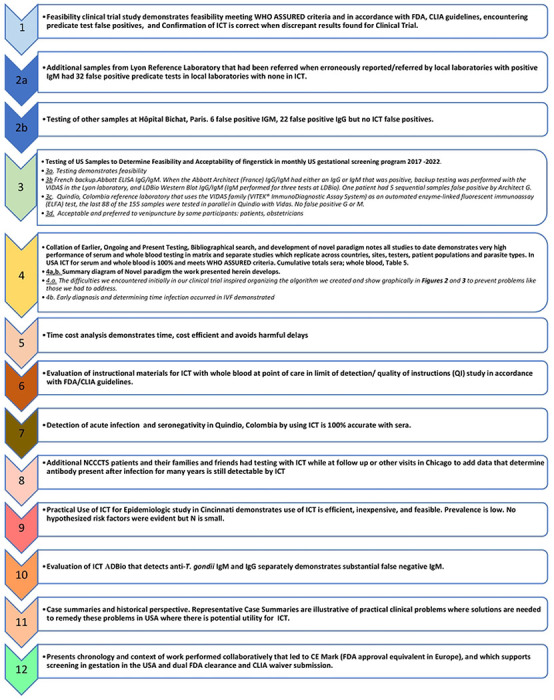
Summary of results of each study.

**Table 1.** Summary of Studies that used LDBIO *Toxoplasma* ICT IgG/IgM (ICT) test on U.S. sera or whole blood samples, with performance results

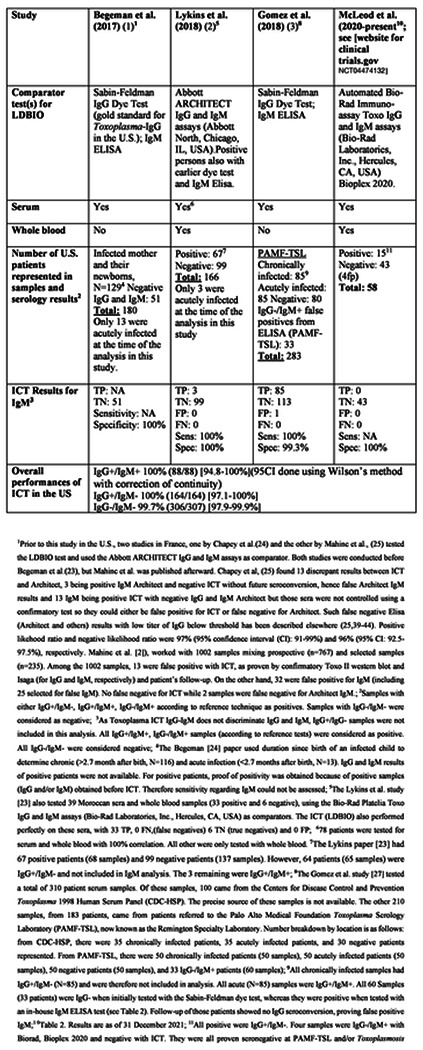

**Table 2.** Comparison of *Toxoplasma* ICT IgG-IgM test and comparator predicate tests that gave false positive results

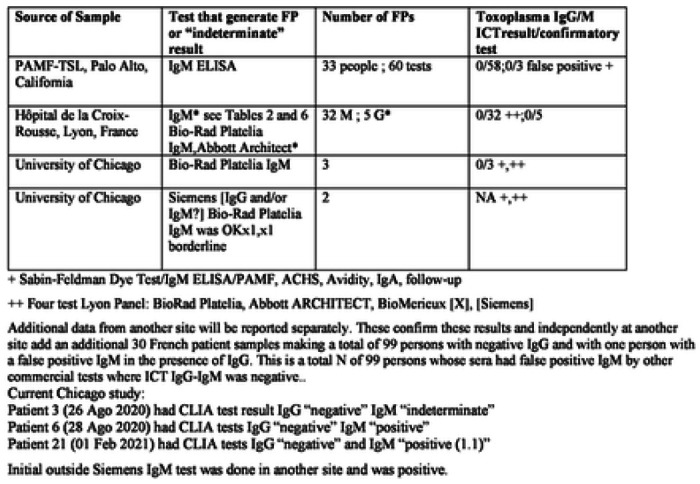

**Table 3.** All studies with ICT

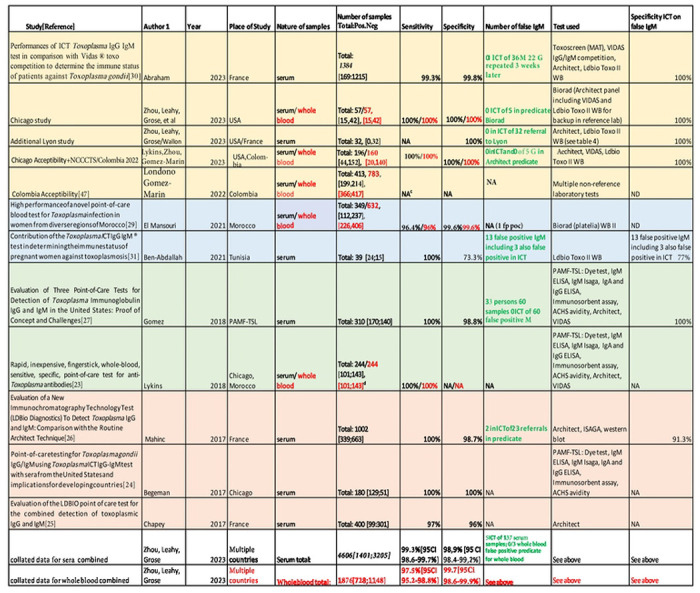

**Table 4.** Approach to screening (also see Supplement Figure S2.1)

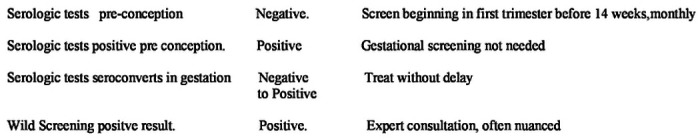

**Table 5.** Cost and Time saving just for fingerprick, all sample handling, reporting, billing for ICT at Point of Care versus Predicate testing

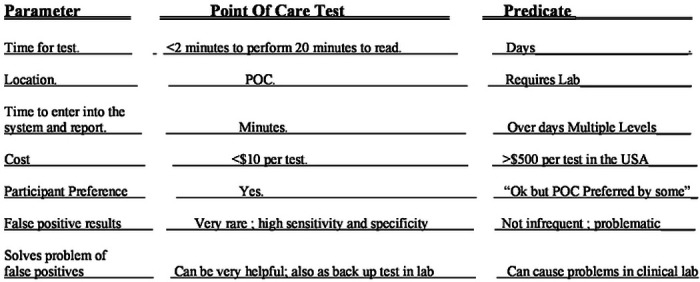

## Data Availability

All data are included in the manuscript and its supplement.

